# Early effects of a high-caloric diet and physical exercise on brain volumetry and behavior: a combined MRI and histology study in mice

**DOI:** 10.1007/s11682-016-9638-y

**Published:** 2016-10-12

**Authors:** Markus Sack, Jenny N. Lenz, Mira Jakovcevski, Sarah V. Biedermann, Claudia Falfán-Melgoza, Jan Deussing, Maximilian Bielohuby, Martin Bidlingmaier, Frederik Pfister, Günter K. Stalla, Alexander Sartorius, Peter Gass, Wolfgang Weber-Fahr, Johannes Fuss, Matthias K. Auer

**Affiliations:** 1 RG Translational Imaging, Department Neuroimaging, Central Institute of Mental Health, Medical Faculty Mannheim, University of Heidelberg, Mannheim, 68159 Germany; 2 RG Clinical Neuroendocrinology, Max Planck Institute of Psychiatry, Kraepelinstrasse 2-10, 80804 Munich, Germany; 3 RG Molecular Neurogenetics, Department Stress Neurobiology and Neurogenetics, Max Planck Institute of Psychiatry, Kraepelinstrasse 2-10, 80804 Munich, Germany; 4 Endocrine Research Unit, Medizinische Klinik und Poliklinik IV, Klinikum der Universität, Ludwig-Maximilians University, Munich, 80336 Germany; 5 Department of Nephropathology, Institute of Pathology, Universitätsklinikum Erlangen, Friedrich-Alexander-Universität Erlangen-Nürnberg, Erlangen, 91054 Germany; 6 RG Animal Models in Psychiatry, Department of Psychiatry and Psychotherapy, Central Institute of Mental Health, Medical Faculty Mannheim, University of Heidelberg, Mannheim, 68159 Germany; 7 Department of Psychiatry and Psychotherapy, Center of Psychosocial Medicine, University Medical Center Hamburg-Eppendorf, 20246 Hamburg, Germany; 8 Institute for Sex Research and Forensic Psychiatry, Center of Psychosocial Medicine, University Medical Center Hamburg-Eppendorf, 20246 Hamburg, Germany

**Keywords:** Obesity, Exercise, Diabetes, Voxel-based morphometry, Cognition, White matter, Grey matter

## Abstract

Excessive intake of high-caloric diets as well as subsequent development of obesity and diabetes mellitus may exert a wide range of unfavorable effects on the central nervous system (CNS) in the long-term. The potentially harmful effects of such diets were suggested to be mitigated by physical exercise. Here, we conducted a study investigating early effects of a cafeteria-diet on gray and white brain matter volume by means of voxel-based morphometry (VBM) and region-of-interest (ROI) analysis. Half of the mice performed voluntary wheel running to study if regular physical exercise prevents unfavorable effects of a cafeteria-diet. In addition, histological analyses for myelination and neurogenesis were performed. As expected, wheel running resulted in a significant increase of gray matter volume in the CA1–3 areas, the dentate gyrus and stratum granulosum of the hippocampus in the VBM analysis, while a positive effect of the cafeteria-diet was shown for the whole hippocampal CA1–3 area only in the ROI analysis, indicating a regional volume effect. It was earlier found that hippocampal neurogenesis may be related to volume increases after exercise. Interestingly, while running resulted in a significant increase in neurogenesis assessed by doublecortin (DCX)-labeling, this was not true for cafeteria diet. This indicates different underlying mechanisms for gray matter increase. Moreover, animals receiving cafeteria diet only showed mild deficits in long-term memory assessed by the puzzle-box paradigm, while executive functioning and short term memory were not affected. Our data therefore highlight that high caloric diet impacts on the brain and behavior. Physical exercise seems not to interact with these mechanisms.

## Introduction

Obesity represents one of the most rapidly growing health burdens in modern societies and sequelae of this development may already be present in children and younger adults (Sinha et al. 2002). It is primarily attributable to the excessive intake of high-caloric meals and a steady decline in physical activity (Lahti-Koski et al. 2002). In the long-term, particularly the development of diabetes contributes to the emergence of micro- and macro-vascular comorbidities (Sullivan et al. 2005). It has been reported that type 2 diabetes mellitus (T2DM) is associated with deficits in learning and memory, attention as well as impaired information processing and executive functions (Cheng et al. 2012). In this context it is striking that cognitive dysfunction has a high prevalence in the diabetic population which may affect up to 17.5 % of subjects (Bruce et al. 2003) and can already be seen in middle aged adults (Ryan and Geckle 2000). Mild cognitive impairment is the dominant finding in diabetic patients and there is evidence that also the risk for the development of dementia is increased. Mild cognitive impairment often represents a transition state in the development of Alzheimer disease (AD) which in turn may contribute to the elevated mortality in diabetic patients (Ahtiluoto et al. 2010). The exact underlying mechanism for this phenomenon has not yet been elucidated, though animal studies indicate that high-caloric diets may negatively affect structure and function of the hippocampus, a brain region critically involved in learning and memory (Stranahan et al. 2008). It has been shown that even in healthy individuals without T2DM, there is shrinkage of the hippocampus in those with glucose levels in the upper normal range (Cherbuin et al. 2012). The underlying mechanisms of diabetic encephalopathies seem to be complex and may be attributable to a variety of factors such as insulin resistance, hyperinsulinemia and hyperglycemia resulting in vascular damage, brain atrophy, and disturbed brain metabolism (Auer et al. 2015). Thus, more research on the mechanisms contributing to cognitive decline in T2DM is warranted. In particular, the understanding of early disturbances in brain integrity should be improved when they might still be reversible and subject to targeted interventions.

Exercise is an important factor for reducing peripheral risk factors of obesity and diabetes such as dysbalanced energy metabolism, glucose use and insulin sensitivity (Cotman et al. 2007). Voluntary exercise may further restore the neuroplastic potential of the brain (Wu et al. 2007). Exercise is thus uniquely positioned to enhance brain health and function by reducing peripheral risk factors and to directly affect brain integrity.

Progress in MR imaging techniques has led to a growing number of primarily human studies investigating structural cerebral changes in metabolic diseases such as T2DM and obesity (Moulton et al. 2015a; Sima 2010).

In particular voxel-based morphometry (VBM) has been widely used to detect changes in brain morphology in a variety of diseases which are accompanied by neurological dysfunction including mild cognitive impairment (MCI) (Karas et al. 2004).

In humans, increased age and body mass index (BMI) can be associated with a decrease in brain volume in general (Ward et al. 2005) and it has been further demonstrated that particularly focal gray matter in the frontal lobe (Pannacciulli et al. 2006) and also in the hippocampus (Moulton 2015a, Raji et al. 2010) is reduced in obese subjects. The degree of glucose tolerance disorders (Samaras et al. 2014) and abdominal obesity rather than total BMI (Debette et al. 2014) have been implicated as potential confounders bringing the metabolic syndrome into focus. Distinct studies in animals in this regard are sparse, though they would provide a chance to get a deeper insight in the contributing factors by experimentally dissecting confounding variables such as diet, glucose intolerance and physical activity. The effects of physical activity on brain volume in general and the hippocampus by MRI volumetry have been studied more intensively in humans (Colcombe et al. 2006; Erickson et al. 2010; Erickson et al. 2011) than in rodent models (Biedermann et al. 2016; Fuss et al. 2010b; Fuss et al. 2014) but studies investigating in parallel MRI volumetrics and underlying histological correlates are sparse. We have shown before that in voluntary wheel running mice hippocampal gray matter volume is highly correlated to the rate of hippocampal neurogenesis (Biedermann et al. 2016). We have also reported that a high caloric cafeteria diet resulted in obesity and the induction of a diabetes-like phenotype which was accompanied by significant changes in hippocampal metabolite levels assessed by 1H magnetic resonance spectroscopic, partially counteracted by physical exercise (Auer et al. 2015). In the present analysis we aimed to investigate how short-term consumption of this diet affects brain volumes. We focused on the hippocampus since it is involved both in cognitive and affective processing and has been suggested as a critical region explaining for cognitive decline in metabolic syndrome (Rosi et al. 2009). We analyzed if physical activity by voluntary wheel running would counteract potentially negative effects on brain volumes. On a cellular level, we hypothesized that the different diets may affect neuron viability, neurogenesis and myelination patterns. As a secondary outcome, we investigated if potential early hippocampal volume changes using VBM analysis would be associated with cognitive deficits in mice reflecting mild cognitive impairment.

## Materials and methods

**Fig. 1:**
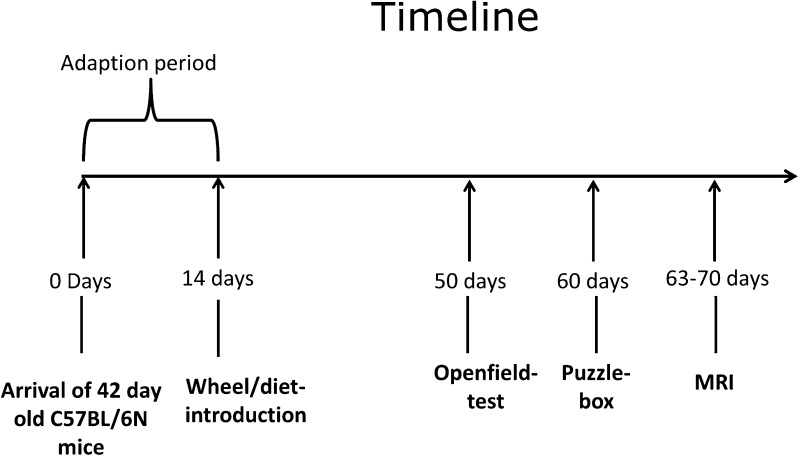
Timeline

All animal experiments were conducted according to the German federal animal welfare legislation and had been approved by the German animal welfare authorities (Regierungspräsidium Karlsruhe, Germany). All institutional and national guidelines for the care and use of laboratory animals were followed. A total of 48 C57BL/6 N male mice obtained at the age of 6 weeks from Charles River (Sulzfeld, Germany) were used to assess the effect of a high-caloric cafeteria diet in running and sedentary mice (Fig. 1).

Details on housing conditions and diet composition have been reported previously as well as other data from the same cohort of mice (Auer et al. 2015). Briefly, mice were kept single-housed in cages with a size of 825 cm^2^ in a temperature and humidity controlled room, on a 12 h light-dark cycle with lights on at 7 p.m. Running wheels equipped with a digital dynamometer were introduced after two weeks and blocked in the sedentary group as described earlier (Fuss et al. 2010a). Running distance was documented on a daily basis. Water and food were available ad libitum. Simultaneously to wheel introduction half of the mice received a high-caloric, cafeteria diet rich in saturated fatty acids and sugars in addition to standard chow (ssniff© R/M-H Extrudat, ssniff Spezialdiäten GmbH, Soest, Germany). For details please see (Auer et al. 2015). Bodyweight was assessed once-weekly in non-fasting animals during the dark-phase.

Accordingly, four experimental groups were defined: standard chow sedentary (SS, n = 12), standard chow runners (SR, n = 12), cafeteria diet sedentary (CS, n = 12) and cafeteria diet runners (CR, n = 12). During the study period two mice died, one due to unknown reasons and one during anesthesia. We therefore had inhomogeneous group sizes (SS = 12, SR = 12, CS = 11, CR = 11). MR volumes of non-fasting mice were acquired after 9–10 weeks of running and diet consumption in a 9.4 T horizontal bore animal scanner equipped with a cryogenic mouse brain coil (Bruker, Ettlingen, Germany).

## Methods

### MR measurements

MR data were acquired in a 9.4 T horizontal bore animal scanner (Bruker, Ettlingen, Germany) equipped with a two element anatomically shaped cryogenic mouse surface coil cooled to 28 K. The cryogenic coil gives an improvement factor of 2.5 to 3.5 in signal to noise ratio compared to the conventional setup using a 4 channel receiver array and a volume transmit coil (Sarah Biedermann et al. 2012). Mice were anesthetized by a gas mixture of O2: 50 % and air: 50 % with ~1.5 % isoflurane. Respiration rate was monitored throughout the experiment. Body temperature was monitored by a rectal thermosensor and maintained at 36 °C by warm water circulation and an external coil-heater.

#### Voxel-Based Morphometry (VBM)

High resolution 3D structural images were acquired using a T2-weighted RARE sequence (Rapid Acquisition with Refocused Echoes, RARE factor 16) with a resolution of 225 × 192 × 96 μm3 at TE = 62.5 ms and TR = 1.2 s (measurement time: 23 min). A total of 46 mice (SS = 12, SR = 12, CS = 11, CR = 11) were measured and included in the VBM analysis. After acquisition, the structural images were resized, coregistered, skull stripped (FSL BET, Smith et al. 2004) and bias-corrected (10 mm cutoff). To account for brain differences, an affine normalization in SPM8 (Wellcome Department of Cognitive Neurobiology, University College of London, UK) was applied. A template of tissue classification maps (gray matter (GM), white matter (WM) and cerebrospinal fluid (CSF) created from 3D images (Biedermann et al. 2012) was used as priors to run an individual segmentation, and the resulting segmented images were smoothed with a 4 mm Gaussian kernel filter.

#### VBM statistics

A full-factorial model in SPM8 was applied to analyze voxel-wise the 3D smoothed data including all groups. The statistics were performed for GM and WM maps using running and cafeteria-diet as factors.

#### ROI analysis

In addition to the voxel-based approach a region-of-interest (ROI) analysis was applied. Binary mask images covering bilateral ROIs including the hippocampal areas CA1–3, dentate gyrus, and stratum granulosum of hippocampus were chosen on basis of the hypotheses and VBM results and created from a labeled anatomical mouse brain atlas (Dorr et al. 2008). These masks were used to create individual ROI images of the affine normalized Jacobi determinant-modulated GM maps. Afterwards, these ROI images were summed up voxel-wise and accounted for the actual measured voxel size resulting in volume estimates given in mm^3^ for each ROI. Statistical analysis of GM ROI volumes was performed using IBM SPSS Statistics for Windows, Version 21.0 (SPSS Inc., Chicago, IL, USA) by applying a multivariate general linear model (GLM) with running and cafeteria-diet as factors.

#### Tissue sampling

For tissue sampling, mice were anaesthetized by i.p. injection of ketamine and xylazine. Mice were perfused transcardially through the left ventricle of the heart with cooled sodium chloride. Brains were removed, split into halves and the randomly selected right and left hemisphere were postfixed for ~8 h in 4 % paraformaldehyde, and kept in phosphate buffered saline (PBS) overnight. 33 μm coronal sections were cut on a vibratome and kept at 20 °C in cryoprotection solution until further processing. Intra-abdominal fat pads were dissected according to the method of Johnson and Hirsch (Johnson and Hirsch 1972).

#### Measurement of blood glucose and insulin levels

For determination of fasting-glucose, mice were food deprived for 6 h before testing. Measurement of blood glucose took place at least 24 h before MRI measurements. Blood was taken from the tail vein and glucose levels were determined using a glucometer (Accu-Chek® Aviva, Roche Diagnostics GmbH). Non-fasting glucose and insulin levels were determined in animals after scarification in ventricular blood. Plasma insulin concentrations were analyzed using commercially available kits (ALPCO, Salem, NH, USA) according to the manufacturer’s instructions.

#### Immunohistochemistry

Every twelfth section of 6–8 animals per group was processed free-floating as described earlier (Auer et al. 2015; Fuss et al. 2010a; Fuss et al. 2010b). To assess the number of newly formed neurons, cells were stained with a primary goat polyclonal anti-DCX-antibody (Doublecortin; 1:1000; sc-8066, Santa Cruz Biotechnology, Santa Cruz, CA, USA). A primary polyclonal rabbit anti-MBP (Myelin Basic Protein)-antibody was used for staining of oligodendrocytes and the myelin sheath (1:300, ab40390, Abcam, Cambridge, MA, USA). Sections were incubated in 0.6 % H2O2 in in Tris-buffered saline (TBS) for 30 min at room temperature to block endogenous peroxidase activity. Sections were preincubated in 2 % normal serum of appropriate species in TBS + 0.2 % Triton™ X-100 (Sigma Aldrich, MO, USA) for 1 h at room temperature and, subsequently, in primary antibody overnight at 4 °C. Sections were rinsed and incubated with biotinylated secondary antibody (rabbit anti-goat IgG; diluted 1:300, VectastainEliteABC kit, Vector Laboratories, Burlingame, CA, USA) for DCX-staining or with Cy3-labeled secondary antibody (1:200, C2821, anti-rabbit IgG–Cy3 antibody, Sigma Aldrich, MO, USA) for MBP visualization. Rinses were performed between all steps using TBS + 0.05 % TritonX-100 (pH 7.4), or TBS (pH 7.4), only. Finally, sections stained for DCX were incubated in avidin–biotin complex (VectastainEliteABC kit) for 20 min at room temperature, and stained using diaminobenzidine (DAB) as chromogen. Slides were finally air dried overnight, dehydrated, coverslipped with Permountor mounting medium. MBP-stained slides were coverslipped with VECTASHIELD Mounting Medium with DAPI (Vector Laboratories Burlingame, CA, USA). Examined was done on a Zeiss Axioskop fluorescence microscope (Carl Zeiss, Oberkochen, Germany).

#### Stereology and morphology

Quantitative analyses were performed as described before (Auer et al. 2015; Fuss et al. 2010a). The average total numbers of DCX-positive cells were estimated using the optical fractionator (West et al. 1991); StereoInvestigator software, MBF Bioscience, Williston, VT). Total numbers of cells were estimated by multiplying the number of cells counted with the inverse of the sampling fraction (West et al. 1991). DCX-positive cells in the hippocampus were counted at 40×. Average cell numbers were calculated by using three sections representing medial, ventral and dorsal hippocampus each. In each of these sections a counting area was applied, separately surrounding the CA1, CA3-region and the dentate gyrus. Cell numbers were calculated by application of a counting grid covering 250 × 250 μm at intervals of 135 μm along the x-axis and 105 μm along they-axis by mean of the optical fractionator. Average cell numbers of each region were calculated by using the mean of three sections (medial, ventral, dorsal hippocampus) of the CA1, CA3, and DG, respectively, and are reported as average cell number per 10,000 μm^2^. For assessment of staining intensity for MBP NIH ImageJ software was used (Jensen 2013). Every picture was taken with the same settings and exposure times. As a region of interest a ventral, dorsal and medial section of the hippocampus was manually outlined and the average intensity was measured. Fluorescence intensity of the hippocampus of a section from the same animal which was only incubated with the fluorescent secondary antibody was then subtracted to determine fluorescence intensity attributable to the primary antibody alone.

#### Behavior

##### Puzzle box

**Fig. 2:**
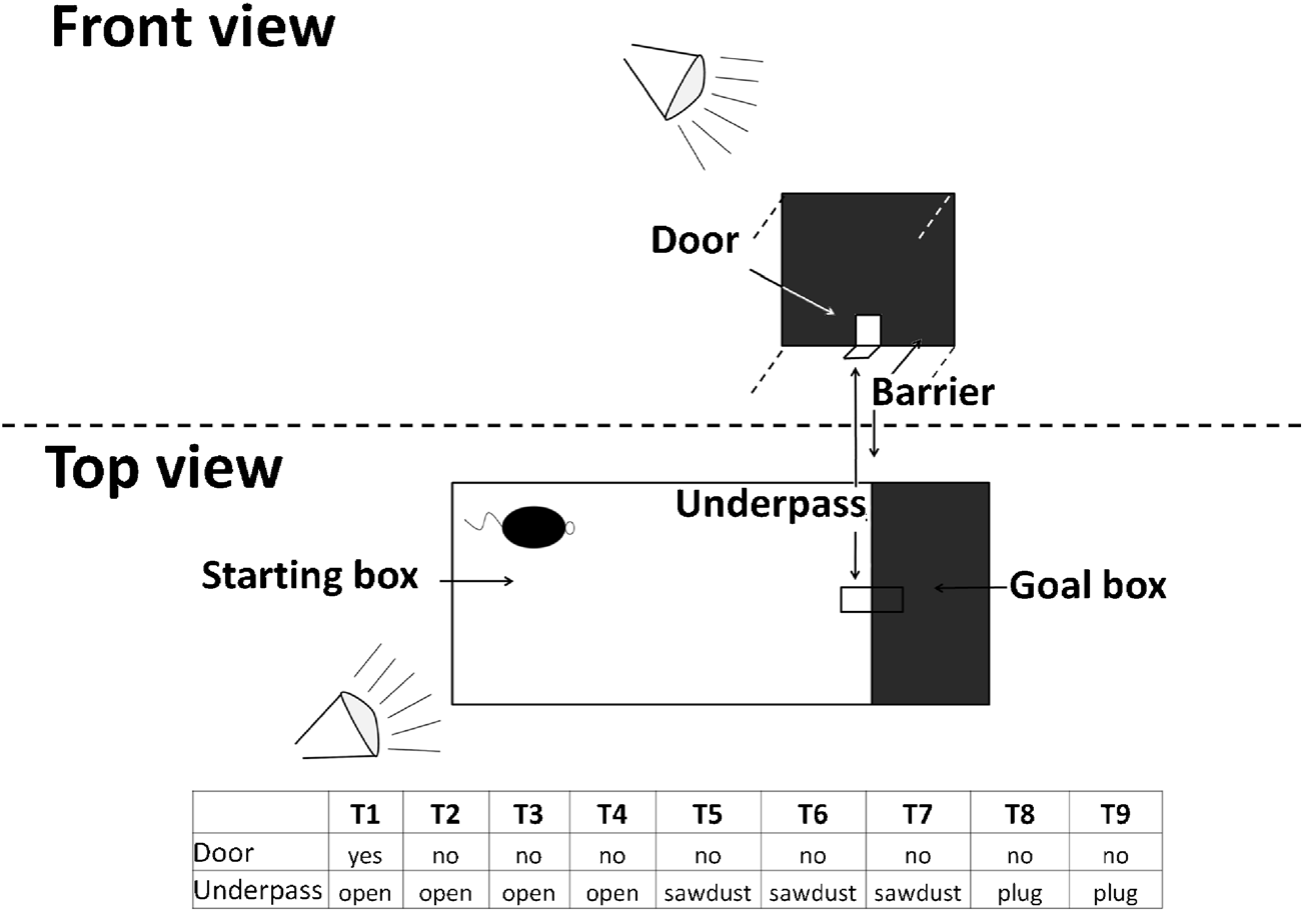
Scheme of the puzzle box test

The puzzle box was used for evaluation of cognitive functioning and has been extensively described previously (Ben Abdallah et al. 2007;). Briefly, mice were put in a container made of Plexiglas subdivided by a removable barrier into a brightly-lit start zone (58 cm long, 28 cm wide) and a smaller covered goal zone (15 cm long, 28 cm wide) (Fig. 2). The test was performed sixty days after wheel introduction. Mice were trained to move into the goal zone through a narrow underpass located under the barrier. Mice underwent a total of nine trials (T1–T9) over three consecutive days, with three trials per day, during which they were challenged with obstructions of increasing difficulty placed at the underpass (T1 = open door, T2/3/4 = open underpass, T5/6/7 underpass filled with sawdust, T8/9 = underpass blocked by removable plug). The puzzle box tests executive functioning by evaluating cognitive flexibility and problem solving strategies (T1,T2,T5,T8) as well as learning and short and long-term memory functioning. Short-term memory can be assessed as every task, which is newly introduced on one day (T2,T5,T8), is once repeated on the same day (T3, T6, T9) and long-term memory is tested as the last test of each day is the same as the first test of the next day (T4, T7). T1 also serves as habituation. Performance of mice in the puzzle box was assessed by measuring the latency to enter the goal zone in seconds.

##### Open field test

The open field test was performed to assess locomotion and anxiety behavior which can influence the results of the puzzle box. Fifty days after wheel introduction mice were tested in the open field apparatus*.* Activity monitoring was conducted in a square shaped, white open field, measuring 50 × 50 cm^2^ and illuminated from above with about 50 Lux as described earlier. The mice were placed individually into the middle of the arena allowed to explore the open field for 10 min. Data was recorded and analyzed using the imaging and video tracking system EthoVision 3.0 (Noldus Information Technology, Wageningen, the Netherlands). Parameters assessed for the present studies were total distance moved and time spent in center.

#### General statistics

Statistical analysis of data on behavior and histology was performed using IBM SPSS Statistics for Windows, Version 21.0 SPSS 21.0 (SPSS Inc., Chicago, IL, USA). All data are reported as means ± s.e.m. Two-factorial analysis of variance (ANOVA) was used to detect differences between the groups. Bonferroni’s post hoc analysis was applied with regard to subgroup differences. Significance was evaluated at a probability of 5 % or less.

## Results

### Effects of diet and running on glucose and insulin levels

As reported before, diet significantly affected fasting glucose levels (F(1,42) = 83.68, *p* < 0.001), while running had no effect in this regard. Fasting glucose in SR animals (115.8 ± 4.8 mg/dL) were not significantly different from SS animals (116.3 ± 5.8 mg/dL). Highest glucose were seen in CS animals (160.1 ± 6.9 mg/dL) followed by CR mice (146 ± 5.4 mg/dL). The cafeteria groups did not differ significantly (*p* = 0.683), while fasting glucose of cafeteria-diet were significantly higher than in animals receiving standard chow (CR versus (SR and SS), *p* = 0.003; CS versus (SR and SS), *p* < 0.001).

There was a significant effect of diet (F(1,20) = 8.23, *p* = 0.011) and running (F(1,20) = 4.5, *p* = 0.05) on insulin levels. Post-hoc analysis revealed that CS animals (0.9 ± 0.27 ng/ml) had significantly higher insulin levels than SR (0.3 ± 0.05 ng/ml) mice (*p* = 0.032), while there was only a trend for higher insulin levels in CS vs. CR mice (0.43 ± 0.05 ng/ml) (*p* = 0.061). SS and SR mice did not significantly differ in this regard (both 0.3 ± 0.05 ng/ml).

### Effects of cafeteria diet and wheel running on brain volume and structure

#### VBM

**Fig. 3:**
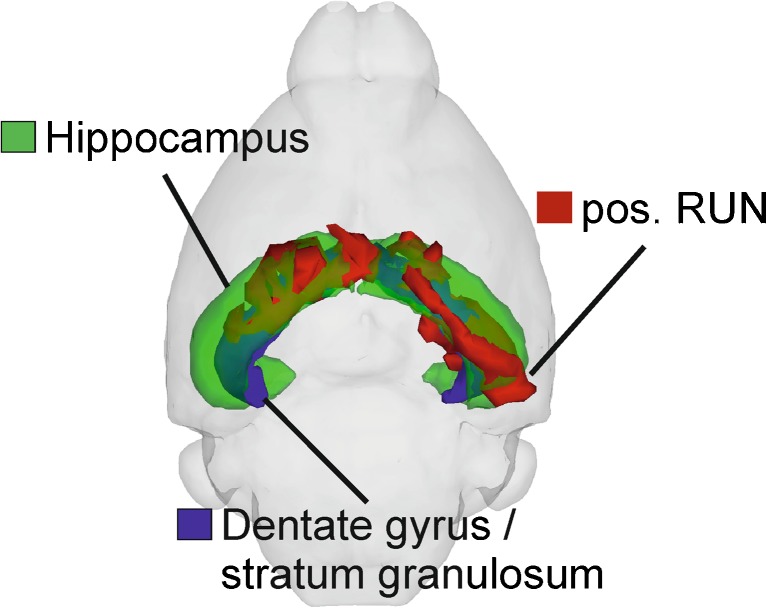
Positive effect of factor running (pos. RUN) depicted in red covering partly the bilateral hippocampal areas CA1–3 (in green), dentate gyrus and stratum granulosum (both in blue)

The voxel-wise VBM analyses yielded in two significant clusters (*p* < 0.001 uncorrected; *T* = 3.031) for a positive effect of running on GM which survive a FWE correction on cluster-level with pFWE <0.001 consisting of 4441 and 2913 voxel, respectively. Within these clusters 247 voxel were found which are significant (FWE-corrected, pFWE <0.019). The two clusters cover partly the hippocampal CA1–3 areas, dentate gyrus, and stratum granulosum of hippocampus as illustrated in Fig. 3. Furthermore, there were no other significant effects after whole brain cluster correction, neither any negative effects of running on GM, nor any effects of cafeteria-diet or interactions on GM and WM maps, respectively.

### ROI analysis

The multivariate GLM, which included the hippocampal CA1–3 areas, dentate gyrus, and stratum granulosum of hippocampus yielded in a highly significant effect of factor running (*p* < 0.001) and in a tendency for factor cafeteria-diet (*p* = 0.087). In the test of between-subjects effects running lead to highly significant results in all anatomical regions investigated (all *p* < 0.001). Whereas, cafeteria-diet showed a significant positive effect only on the hippocampal areas CA1–3 (*p* = 0.017) in the test of between-subjects effects, which would survive correction for multiple comparison up to a factor of 2. There was no significant correlation between average daily running distance and hippocampal volume (CC 0.049; *p* = 0.848).

### To determine if differences in gray matter were due to increased neurogenesis we analyzed the number of DCX-expressing cells in the hippocampus by ex vivo immunohistochemistry

**Fig. 4:**
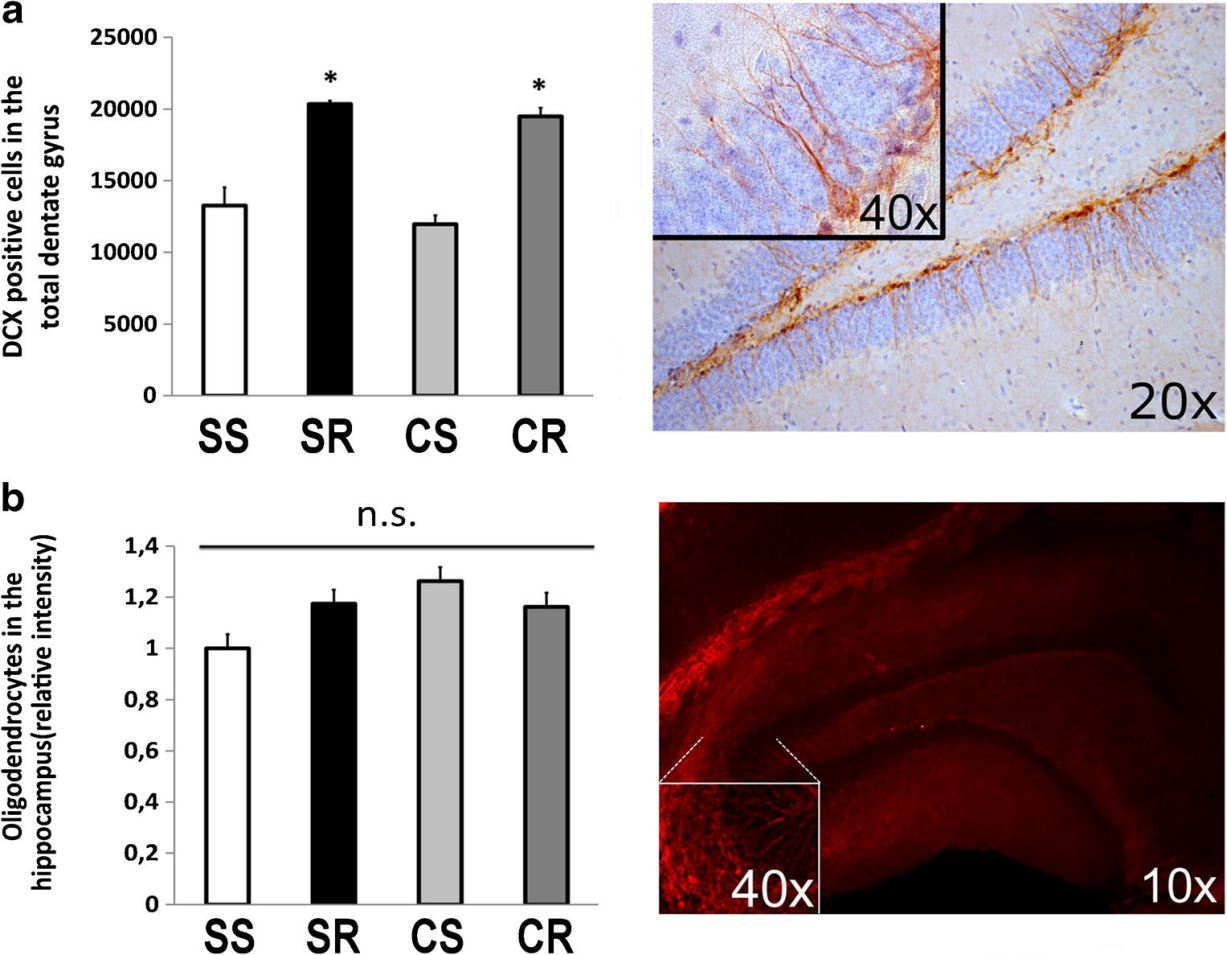
Long-term wheel running within the observation-period had a significant effect on doublecortin-positive cells (DCX) in the subgranular zone of the dentate gyrus (F_1,19_ = 8.33; *p* = 0.009) irrespective of the diet provided. In post-hoc-analysis, CR animals had a higher number of DCX-positive cells (19,496 ± 812) in the hippocampus than did CS (11,957 ± 851; *p* < 0.001) and SS animals (13,258 ± 1500; *p* = 0.001). Number of DCX- positive cells in SR (20,358 ± 488) animals was higher than in the SS group (*p* = 0.002) (**a**). There was neither an effect of diet nor of running on mean intensity for MBP fluorescent staining (**b**). * indicates a significant difference between SR and CR vs SS and CS

Indeed, long-term wheel running within the observation-period had a significant effect on doublecortin-positive cells (DCX) in the subgranular zone of the dentate gyrus (F_1,19_ = 8.33 *p* = 0.009) irrespective of the diet provided. In post-hoc-analysis, CR animals had a higher number of DCX-positive cells (19,496 ± 812) in the hippocampus than did CS (11,957 ± 851; *p* < 0.001) and SS animals (13,258 ± 1500; *p* = 0.001). Number of DCX- positive cells in SR (20,358 ± 488) animals was higher than in the SS group (*p* = 0.002) (Fig. 4a). As opposed to these results there was neither an effect of diet nor running on mean intensity for MBP fluorescent staining (Fig. 4b).

### Effects of diet and exercise on learning and memory

To test the functional consequences of changes to brain volume and neurogenesis, we tested mice in the puzzle box test.

### Puzzle box

**Fig. 5:**
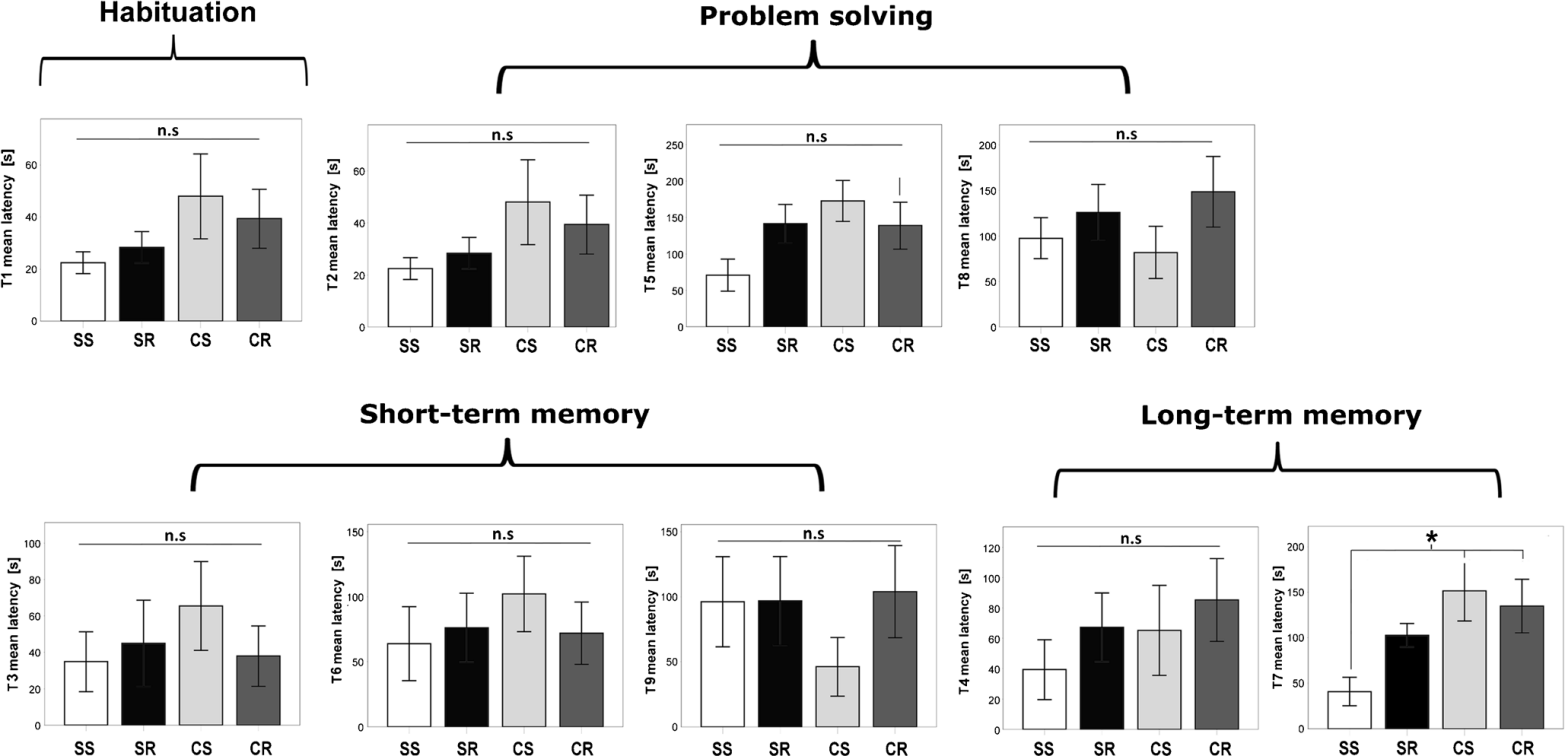
Neither cafeteria diet nor running had an effect on the latency time for goal entering of the different parts of the puzzle box paradigm. Group assignment did not impact measures of cognitive flexibility (T2, T5, T8) nor short-term memory (T3, T6, T9). There was a significant positive effect of cafeteria diet but not for running on time to solve task number T7 (F_1,46_ = 9.086; *p* = 0.004), indicating some long-term memory deficits, though this was not true for T4. In post-hoc-analysis, SS (40,9 s ± 15.5; *p* = 0.046) mice needed significantly less time to leave the start zone than CS (151.5 s ± 33.2) and CR (97.8 s ± 29.5) mice (*p* = 0.012), while there was no significant difference between SS and SR mice (102 s ± 13.1). There was no indication of increased anxiety, as determined by “time in the center zone” (d)

Neither cafeteria diet nor running had an effect on the latency time for goal entering of the different parts of the puzzle box paradigm. Group assignment did not impact measures of cognitive flexibility (T2, T5, T8) nor short-term memory (T3, T6, T9). There was a significant positive effect of cafeteria diet but not for running and time to solve task number T7 (F_1,46_ = 9.086; *p* = 0.004), indicating some long-term memory deficits, though this was not true for T4. In post-hoc-analysis, SS (40.9 s ± 15.5; *p* = 0.046) mice needed significantly less time to leave the start zone than CS (151.5 s ± 33.2) and CR (97.8 s ± 29.5) mice (*p* = 0.012), while there was no significant difference between SS and SR mice (102 s ± 13.1) (Fig. 5). There was no significant difference between the groups for the time to reach the goal (data not shown).

### Open field test and overall activity

**Fig. 6:**
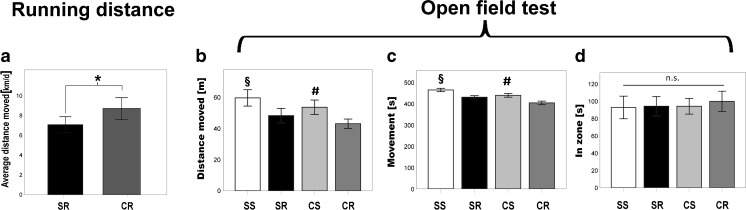
These results did not seem to be confounded by locomotion, since the average daily running distance in SR animals with 7.1 km/day (± 0.4) was lower in comparison to 8.7 km/day ±0.5 in CR animals (*p* = 0.025) (**a**). Although cafeteria diet fed mice were moreover less active in the open field test in distance travelled (F_1,46_ = 6.31; *p* = 0.016) and movement time (F _1,46_ = 10.63; p = 0.002) (a + b) in comparison to sedentary controls. The overall activity of wheel running mice was also reduced. Running mice traveled significantly shorter distances in the arena compared to sedentary controls (F_1,46_ = 24.29; p < 0.001) (**b**). Correspondingly, the time of movement was lower in runners (F_1,46_ = 18.81; p < 0.001) (**c**). There was no indication of increased anxiety, as determined by “time in the center zone” (**d**) (F_1,46_ = 0,1; *p* = 0.76).§ indicates a significant difference between SS vs. CR and SR. # indicates a significant difference between CS vs. SR and CR

These results did not seem to be confounded by locomotion, since the average daily running distance in SR animals with 7.1 km/day (± 0.4) was lower in comparison to 8.7 km/day ±0.5 in CR animals (*p* = 0.025) (Fig. 6a). Cafeteria diet fed mice were moreover less active in the open field test in distance travelled (F_1,46_ = 6.31; *p* = 0.016) and movement time (F_1,46_ = 10.63; *p* = 0.002) (Fig. 6a+b) in comparison to sedentary controls. The overall activity of wheel running mice was also reduced. Running mice traveled significantly shorter distances in the arena compared to sedentary controls (F_1,46_ = 24.29; *p* < 0.001) (Fig. 6b). Correspondingly, the time of movement was lower in runners (F_1,46_ = 18.81; p < 0.001) (Fig. 6c). There was no indication of increased anxiety, as determined by “time in the center zone” (d) (F_1,46_ = 0,1; *p* = 0.76) (Fig. 6c).

## Discussion

Our hypothesis was that a cafeteria-diet may impair hippocampal plasticity and thus affect hippocampus-dependent behavior. In the present study, we found that although cafeteria diet was inducing a strong metabolic syndrome-like phenotype (Auer et al. 2015), it did not result in shrinkage of hippocampal GM volume but instead resulted in a GM increase as found by the ROI analysis. Correspondingly, hippocampal dependent behavior was only mildly affected by diet.

Interestingly, the ROI analysis yielded in a tendency of higher GM in the investigated regions by cafeteria-diet which was significant in the hippocampal CA1–3 area whereas VBM showed no effect for cafeteria-diet. Due to its voxel-based character VBM is more sensitive for localized changes which affect the same location (or voxels) in every subject whereas the ROI analysis can reveal changes affecting whole brain regions. This indicates that cafeteria-diet has possibly a rather regional effect on the whole hippocampal CA1–3 area which does not affect the same location within this region in every subject and would explain why the ROI analysis yielded in a cafeteria-diet effect and VBM did not.

Our finding is thus in contrast to several studies in humans with T2DM that have found GM decline in the hippocampus (Kamiyama et al. 2010; Moran et al. 2013; Weinstein et al. 2015) as well as disturbances in WM (Xiong et al. 2016; Wang et al. 2014). Moran et al. (2013) reported a decline in various cognitive domains that was primarily explained by reduction of hippocampal and total GM but independent of WM alterations or the degree of cerebrovascular lesions (Moran et al. 2013). In line with the fact that we did not observe shrinkage in any brain volume measurement, cafeteria diet did per se not result in decline in executive or short-term memory functions determined by the puzzle box paradigm, though there was a mild negative effect on long-term memory. This may be due to the relatively short period of exposure to the diet and hence the limited negative effects on structural variables. Other authors also suggested that GM shrinkage in T2DM may also be due to hypoperfusion and not structural changes as they are to a large extent reversible upon initiation of insulin therapy (Chen et al. 2014).

An explanation might be that though there have been reports on a positive association of high-normal glucose levels in diabetes-free subjects of the general population with hippocampal atrophy (Weinstein et al. 2015), most studies indicate that these changes are primarily seen in those patients with longer disease duration and higher HbA1c levels (Ebady et al. 2008; Lin et al. 2013) and those with late sequelae such as micro- or macrovascular damage (Wessels et al. 2006, Moulton et al. 2016) and peripheral neuropathy.

Interestingly, in contrast to the effect of running, cafeteria diet did not affect the stratum granulosum (ROI analysis) which is in accordance to our histological evaluation revealing a significant increase in newly formed neurons identified by DCX staining only by running but not by cafeteria diet.

Neurogenesis in adulthood has in particular been shown to occur in the DG of the hippocampus and the subventricular zone (SVZ) (Ming and Song 2011). While voluntary wheel running is a well-established factor, positively influencing hippocampal neurogenesis in rodents (Fuss et al. 2010a; Van Praag et al. 2005) others have shown that high caloric diet paradims can result in a decrease in new-born neurons (Boitard et al. 2012; Park et al. 2010) as well as in a decrease of brain-derived neurotrophic factor (BDNF) (Molteni et al. 2002; Pistell et al. 2010). The underlying mechanism may be multifactorial and reach from diet-induced central inflammation (Pistell et al. 2010) involving central cornerstones of inflammatory pathways such as NF-κB (Li et al. 2012; Vinuesa et al. 2016) to overfeeding-driven central insulin resistance affecting hippocampal neuron survival (Stranahan et al. 2008) to an increase in oxidative stress markers (Park et al. 2010). Vice versa, it has been demonstrated that diet restriction may result in increased neurogenesis in the DG via an increased survival rate of new-born neurons (Lee et al. 2002; Hornsby et al. 2016) that has been attributed to the associated increase in BDNF (Hornsby et al. 2016). In a recent study another potential mechanism has been brought into play. Calorie restriction for two weeks that is known to increase levels of the “hunger-hormone” ghrelin as well as direct injections of this hormone resulted in increased neurogenesis in the hippocampus (Hornsby et al. 2016). In line, diet-mediated effects on neurogenesis in the DG were prevented in Ghrelin receptor knock-out mice (Kim et al. 2015) in an earlier study.

Potential explanations why we did not observe differences in hippocampal neuron survival are that this effect may depend on age (Boitard et al. 2012) and diet composition (Strandberg et al. 2008). Importantly, one might further speculate that the constant change in diet including a free choice paradigm with significant differences in taste and structure, may represent a kind of enrichment in our model (Kempermann et al. 1997). This might have counteracted the negative effects of the nutritional content or its metabolic sequelae itself. For example, it has been demonstrated before that enriched conditions not only are capable of affecting hippocampal neuron viability or neurogenesis but also a variety of neuroplastic processes that may contribute to the GM signal (Lerch et al. 2011; Van Praag et al. 2005). However, this is highly speculative and requires further investigation e.g. by comparing a cafeteria diet with a commercially available high-fat diet.

Another possible explanation as to why runners receiving a cafeteria diet in comparison to runners on standard chow did not differ in GM, could be that cafeteria diet did significantly affect running behavior. Mice receiving the high-caloric diet ran on average 22.5 % more than their counterparts on standard chow, an effect which had already been described in mice bred for extensive running behavior before (Meek et al. 2012; Meek et al. 2010). This might have counteracted the negative effects of the diet itself, although there was no direct correlation of average running distance and hippocampal volume.

We have previously shown that this paradigm does not affect either total neuron or microglia count in the stratum granulosum of the hippocampus (Auer et al. 2015), questioning their role in the observed GM change. This indicates that the effect of cafeteria diet on hippocampal volume is due to other potential contributors to hippocampal GM volume such as dendrite spine density, changes in neuronal morphology or vasculature and perfusion (Zatorre et al. 2012).

How obesity or its sequalae such as chronic hyperglycemia in T2DM might affect white matter on a microstructural level is less well understood. WM is composed of bundles of myelinated axons, connecting gray matter areas within in the brain (Fields 2008). The myelin sheath in the brain is formed by oligodendrocytes and myelin consists of lipids and proteins such as MBP. Myelination is a dynamic process across the lifespan (Kochunov et al. 2012) and for most brain regions seems to peak in early-to-middle adulthood (Kochunov et al. 2012). Disturbances in the integrity of WM have been linked to cognitive decline in aging as in T2DM (Bussel et al. 2016) and may involve hypomyelination and/or decreased oligodendrocyte numbers (Bartzokis et al. 2004). Although we did not observe significant changes on WM in VBM and in MBP expression on a histological level in our model, there are different theories linking the metabolic syndrome with white matter alterations. Again central inflammation may be responsible for WM disturbances in chronic overfeeding (Rosenberg 2009). It has been further speculated in this context that high-caloric diets may promote so-called endoplasmic reticulum (ER) stress that is also a central element in inflammatory signaling in obesity and T2DM on a peripheral organ level (Özcan et al. 2004; Hummasti and Hotamisligil 2010). Increased ER stress has further been shown to be involved in myelination abnormalities in neurodevelopmental disorders (Lugar et al. 2016). That chronic overfeeding can result in ER stress on a CNS level has been demonstrated for hypothalamic (Thaler et al. 2012) and hippocampal neurons and also been linked to central insulin resistance (Sims-Robinson et al. 2016). Pathways that are involved in ER stress can be directly activated by nutrients such as glucose and saturated fatty acids (FA) (Wei et al. 2006). It has been suggested that oligodendrocytes would be highly sensitive to ER disruption as the ER is needed to fulfill their need to synthesize large quantities of membrane lipids, proteins and cholesterol, hereby placing them at a particular risk for apoptosis (Lin and Popko 2009).

### Strength and limitations

A strength of our study is that, to our knowledge, it is the first of its kind investigating the effects of a high-caloric diet resulting in a diabetic phenotype in conjunction with a model of physical exercise on brain volumes assessed by VBM analyses in rodents. It is also the first of its kind separately investigating gray and white matter in this setting. We further provided data on the cognitive outcome of these animals as well as on the effects of group assignment on a histological level. A limitation is that for the assessments of food intake and calculation of macronutrient composition it was inevitable to house the animals individually which has been suggested to change behavior and may also affect CNS processes, mainly attributable to impoverishment of the environment. However, our animals were living in large cages with environmental enrichment (bedding material, blocked or free running wheels) to mitigate an effect of impoverishment. Single housing is routinely performed in wheel running studies and it has also been shown that single housing does not necessarily induce changes in any major immuno-endocrine category under non-stressed conditions.

## Conclusion

In contrast to earlier reports, we showed here that a high-fat and high-caloric cafeteria diet may at least in short term have a regional positive effect on hippocampal gray matter volume of mice. While wheel running also increased gray matter volume and hippocampal neurogenesis, the gray matter increase through diet seemed to be mediated independently of hippocampal neurogenesis.
